# Visualizing Cellular Dynamics and Protein Localization in 3D Collagen

**DOI:** 10.1016/j.xpro.2020.100203

**Published:** 2020-12-08

**Authors:** Wan Hon Koh, Romaniya Zayats, Paul Lopez, Thomas T. Murooka

**Affiliations:** 1 University of Manitoba, Rady Faculty of Health Sciences, Department of Immunology, Winnipeg, Canada; 2 University of Manitoba, Rady Faculty of Health Science, Department of Medical Microbiology and Infectious Diseases, Winnipeg, Canada

**Keywords:** Cell Biology, Cell-based Assays, Immunology, Microscopy

## Abstract

Immune cells migrate and communicate through cell-to-cell interactions and cytokines to coordinate the specificity and timing of the immune response. While studying these events in cell culture are standard procedure, spatiotemporal dynamics of cell-to-cell interactions within three-dimensional (3D) environments are critical in generating appropriate effector functions. Here, we present a detailed protocol to study cells within an all-in-one 3D collagen matrix that is amenable to live-cell microscopy and immunohistochemistry. This approach facilitates analyses of dynamic cellular events in 3D settings.

For complete details on the use and execution of this protocol, please refer to Koh et al. (2020).

## Highlights

• Use of 3D collagen chamber to study cell-to-cell interaction in a 3D environment
• Optimized protocol to perform live-cell microscopy and cell migration analysis
• A step-by step immunohistochemistry protocol in collagen to preserve 3D morphology

## Graphical Abstract

**Figure:**
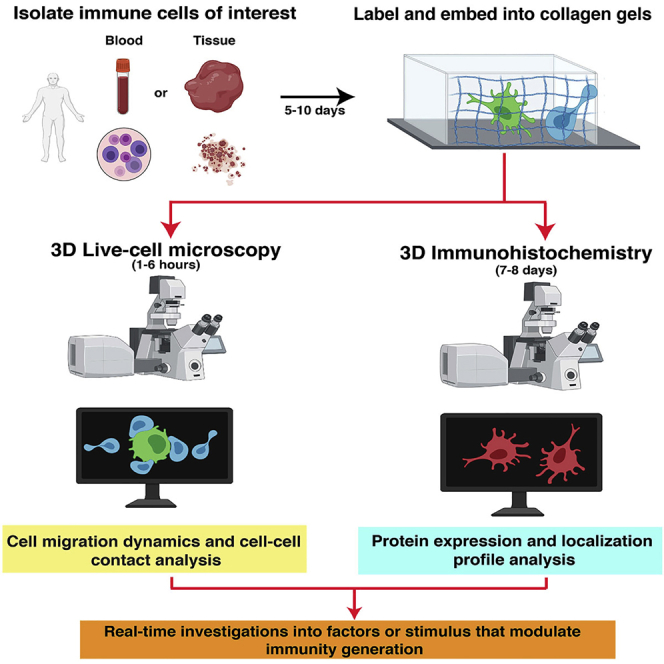

## Before You Begin

### Isolation and Storage of Peripheral Blood Mononuclear Cells (PBMCs)

**Timing: 3–4 h for isolation; can store cells in liquid nitrogen up to 6 months**

1. Dilute fresh buffy coats (40 mL) from healthy donors 1:1 with sterile PBS (40 mL).
2. Add 15 mL of Ficoll-Hypaque solution into four 50 mL conical tubes. Carefully overlay 20 mL of diluted buffy coat onto the Ficoll-Hypaque layer. Avoid mixing of the two layers.
3. Balance the tubes within ±0.2 g and centrifuge for 30 min at 400 × *g* at 20°C–22°C with the brakes turned off.
4. Carefully remove cells from the interface using a transfer pipet and place in a new 50 mL conical tube. Wash cells twice by topping up to 40 mL with complete RPMI media or PBS and centrifuge for 10 min at 500 × *g.*

***Optional:*** To remove residual red blood cells, ACK lysis can be performed at this stage. Resuspend cell pellet in 10 mL ACK lysis buffer and incubate for 5 min at 20°C–22°C.

5. Resuspend the cell pellet in 30 mL of complete RPMI media. Remove any cell clumps. Perform a 1:1 dilution with trypan blue (10 μL of diluted cell suspension with 10 μL of trypan blue) and place 10 μL into the hemocytometer to count cells and record cell viability. Dilute cell suspension if density of cells in the hemocytometer is too high to count accurately.
6. Centrifuge PBMCs for 10 min at 500 × *g.* Resuspend cells with sterile freezing media (50% complete RPMI media, 40% FCS, 10% DMSO) at a final concentration of 50–100 × 10^6^ cells/mL.

**CRITICAL:** Once cells are resuspended in freezing media, work quickly to place cryotubes in the freezer. Cell viability is reduced when in contact with DMSO containing solution for an extended period of time.

7. Pipette 1 mL of cell suspension into a 1.5 mL screw top cryotube, place in a Mr. Frosty cryopreservation container and into the −80°C freezer for 18–24 h.
8. Remove cryotubes and place in liquid nitrogen for long term storage.

***Note:*** Please adhere to institutional biosafety and bioethics regulations for work using human blood products. Donor information should be anonymized and screened for infectious agents, such as HIV and Hepatitis virus.

## Key Resources Table

**Table:** 

REAGENT or RESOURCE	SOURCE	IDENTIFIER
**Antibodies**		

Human CD3 (clone: HIT3a)	Biolegend	Cat #300318
Human CD4 (clone: RPA-T4)	Biolegend	Cat #300537
Human CD11c (clone: 3.9)	Biolegend	Cat #337218
Human CD14 (clone: 63D3)	Biolegend	Cat #367116
Human CD45RO (clone: UCHL1)	Biolegend	Cat #304230
Human CD62L (clone: DREG-56)	Biolegend	Cat #304814
Human CD80 (clone: 2D10)	Biolegend	Cat #305218
Human CD86 (clone: IT2.2)	Biolegend	Cat #305406
Human CCR7 (clone: G043H7)	Biolegend	Cat# 353214
Human HLA-DR (clone: L243)	Biolegend	Cat #307628
anti-human CD3ε/CD28 antibody-coated Dynabeads	Life Technologies	Cat #11131D

**Biological Samples**		

Buffy coats from healthy anonymized donors	Canadian Blood Services (CBS)	n/a

**Chemicals, Peptides, and Recombinant Proteins**		

Human recombinant IL-2	Peprotech	Cat #200-02
Human recombinant IL-4	Biolegend	Cat #574008
Human recombinant GM-CSF	Biolegend	Cat #578606
Ficoll-Hypaque solution	GE Healthcare	Cat #17-1440-03
LPS from *Salmonella Minnesota*	Invivo Gen	Cat #tlrl-smlps
Fetal calf serum (FCS)	VWR Seradigm	Cat #1500-500
Nunclon Sphera flasks	Thermo Scientific	Cat #174951
GlutaMAX Supplement	Gibco	Cat #35050061
Sodium pyruvate (100 mM)	Gibco	Cat #11360070
HEPES (1 M)	Sigma	Cat #H3375-500G
Penicillin/streptomycin solution (10,000 U/mL)	Gibco	Cat #15140122
Tween 20	Sigma	Cat #P9416-100ML
Triton X-100	Sigma	Cat #T8787-50ML
Glycine	Sigma	Cat #G7126-100G
Bovine serum albumin	Sigma	Cat #A8806-5G
10× MEM	Gibco	Cat #11430-030
7.5% sodium bicarbonate solution	Gibco	Cat #25080-094
PureColl bovine collagen solution (3.1 mg/mL)	Advanced Biomatrix	Cat #5005-100ML
Glass microscope slides	Thermo Fisher	Cat #12-550-015
Glass coverslips (18 mm × 18 mm)	VWR	Cat #16004-094
Adhesive silicone isolators (1 mm thick)	EMS	Cat #70336-72
High vacuum grease	Dow Corning	Cat #Z273554

**Critical Commercial Assays**		

Human CD14+ monocyte isolation kit	STEMCELL Technologies	Cat #17858
Human CD4+ T cell isolation kit	STEMCELL Technologies	Cat #17952
EasySep Buffer	STEMCELL Technologies	Cat #20144

**Software and Algorithms**		

Imaris 8.2	Bitplane	n/a
ImarisFileConverter	Bitplane	n/a
ImageJ/Fiji	NIH/freeware	n/a
Prism 8	GraphPad	n/a
FlowJo	Treestar	n/a
MATLAB	MathWorks	n/a
Ultima Multiphoton Microscope	Bruker	n/a

## Materials and Equipment

### Preparation of Media

**Timing: 1 h preparation time**

Complete RPMI 1640 or DMEM media can be prepared beforehand. Each media is supplemented with heat-inactivated 10% fetal calf serum (FCS), and final concentrations of 2 mM GlutaMAX, 1 mM sodium pyruvate, 10 mM HEPES and 1× penicillin/streptomycin. Complete media are stored at 4°C, and warmed to 37°C by placing in a water bath for 30 min prior to working with cells.

**Table:** Bovine Collagen Gels (1.7 mg/mL)

Reagent	Final Concentration	Amount	Storage Condition
10× MEM	0.74×	20 μL	4°C
7.5% sodium bicarbonate	0.28%	10 μL	4°C
3.1 mg/mL Bovine PureColl	1.7 mg/mL	148 μL	4°C
Cells in complete RPMI	Up to 1 million total cells	92 μL	n/a
**Total**	n/a	**270 μL**	n/a

**Table:** Reagents for Preparing Collagen Gels for IHC

Reagent	Amount	Storage condition
4% paraformaldehyde/5% sucrose solution	2 mL	20°C–22°C
Glycine buffer (0.15 M, pH 7)	2 mL	20°C–22°C
0.5% Triton X solution	2 mL	20°C–22°C
1% bovine serum albumin solution	2 mL	4°C
PBS + 0.2% Tween-20 wash solution	100 mL	20°C–22°C

UItima Multiphoton Microscope (Bruker) with two Ti:sapphire lasers (Coherent) with four de-scanned PMT detectors.

***Alternatives:*** A spinning disk or laser scanning confocal microscope can also be used, but these systems may induce more phototoxicity and photobleaching of cells.

Heating platform attached to a temperature controller apparatus (Werner Instruments; TC344C). Humidity or CO_2_ control is not required for this collagen chamber setup.

**Figure 1:**
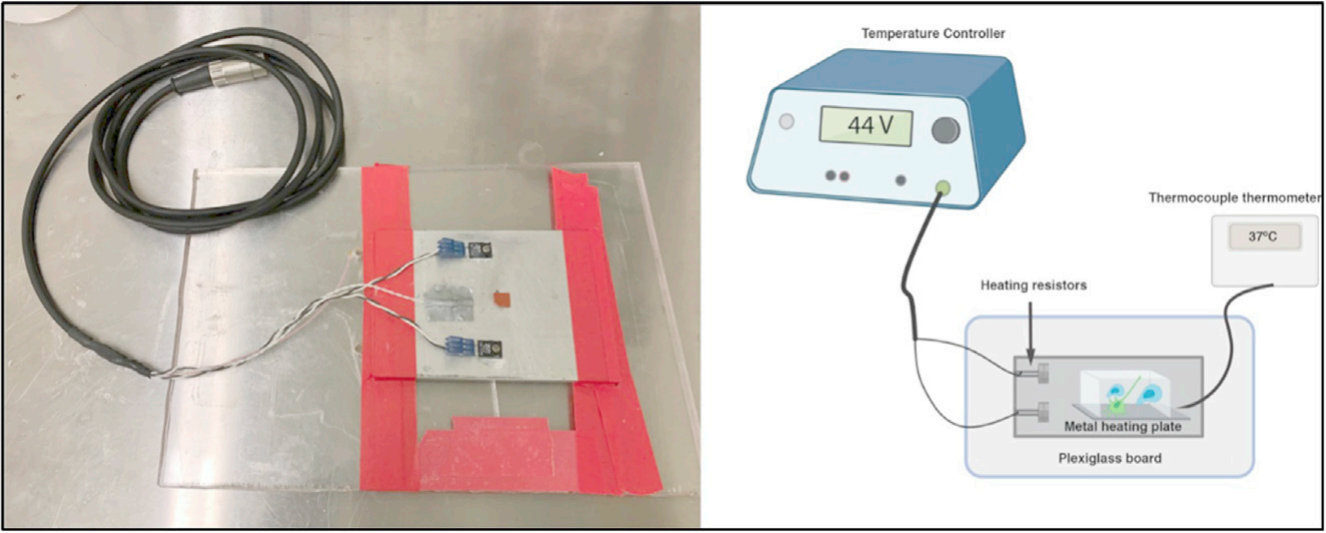
Live-Cell Imaging Set up for Microscopy Left: custom heating platform with two heating resistors to maintain even distribution of heat across the metal platform. Right: setup for live-cell imaging. Heating platform is attached to a temperature controller apparatus and continuously monitored at 37°C using a thermocouple. Illustration was created with BioRender.com.

Thermocouple Thermometer (Cole Parmer #RK-91210-30) with thermocouple probe (Omega #5SC-TT-K-40-72) to continuously monitor the temperature of the collagen chamber (Figure 1).

## Step-By-Step Method Details

### Isolation and Expansion of Immune Cell Subsets from PBMCs

**Timing: 5–10 days to isolate and expand CD4+ T cells**

Cells of interest are isolated and expanded in cell culture. Confirmation of cell purity and subset analysis are determined by flow cytometry.

#### Isolation and Expansion of Activated CD4+ T Cells

1. **Day 0.** Thaw out cryovial(s) of PBMCs by placing them in the 37°C water bath. Typically, 50–100 × 10^6^ cryopreserved PMBCs will yield approximately 2–5 × 10^6^ naive CD4+ T cells. Once partially thawed, add warmed media to the cell suspension and place into a 50 mL conical tube at a 1:9 ratio (1 mL cells:9 mL RPMI medium).

**CRITICAL:** Once cells are resuspended in freezing media, work quickly to place cryotubes in the freezer. Cell viability is reduced when in contact with DMSO containing solution for an extended period of time.

Centrifuge cell suspension for 10 min at 400 × *g* at 20°C–22°C. Decant supernatant and add 10 mL of fresh complete RPMI media. Repeat two times.

2. Count the cells and assess cell viability using trypan blue. Ideal viability at this stage is 80% or higher. Resuspend cells with EasySep Buffer at 50 × 10^6^ cells/mL concentration. Perform negative selection to isolate naive CD4 T cells using the EasySep human naive CD4 T cell isolation kit (STEMCELL Technologies), following manufacturer’s protocol.
3. Decant selected T cells into a 15 mL conical tube and wash with 5 mL complete RPMI medium.
4. Count isolated CD4+ T cells using a hemocytometer and resuspend at 10^6^ cells/mL in complete RPMI medium. Pipette 1 mL of the cell suspension into each well of a 24 well plate (10^6^ T cells per well) and add 25 μL of anti-CD3ε/CD28 antibody-coated Dynabeads and mix gently by pipetting up and down (1:1 beads to cells ratio). Place culture plate in the 37°C/5% CO_2_ incubator for 48 h.
5. **Day 2.** Remove beads by placing cell suspension in a 5 mL Falcon tube and placing it into the EasySep magnet (STEMCELL technologies) for 2 min. Carefully decant T cells into a fresh 15 mL conical tube and wash with 3 mL of complete RPMI media.
6. Count cells and adjust cell concentration to 0.1–0.2 × 10^6^ cells/mL in complete RPMI medium with 8 ng/mL recombinant human (rh) IL-2 into a T25 or T75 flask, standing up. This cell concentration allows T cells to expand over the next 3 days without changing the media.
7. **Day 5.** Change media and re-adjust cell concentration to 10^6^ cells/mL in complete RPMI medium + 8 ng/mL rh IL-2. Adjust the final culture volume according to the number of activated T cells needed for experiments.
8. **Days 6–9.** Perform phenotypic analysis of T cell cultures using flow cytometry, using fluorochrome-tagged anti-CD3, CD4, CD8, CD62L, CD45RO, and CCR7 antibodies to distinguish between effector and central memory T cell subsets. Cells from days 6–9 in culture are typically used for downstream imaging and HIV infection experiments.

***Note:*** The starting number of naive T cells will dictate how many activated cells are obtained at days 6–9. Although T cells undergo exponential proliferation after anti-CD3/CD28 stimulation, ensure that you start with a sufficient naive CD4 T cell numbers to obtain the required number of activated cells for imaging experiments. Activation and expansion of 1 million naive CD4 T cells results in 20–40 × 10^6^ resting memory cells at day 7.

***Note:*** The number of phenotypically central memory (CD45RO^+^CD62L^+^CCR7^+^) and effector memory (CD45RO^+^CD62L^neg^CCR7^neg^) T cells at day 7 can vary between blood donors. It is important to take this into consideration for all downstream imaging studies.

***Note:*** Naive T cells can be directly isolated from fresh PBMCs, typically yielding cells that have high viability.

**Timing: 5–7 days to isolate and culture MDDCs**

#### Isolation and Differentiation of Monocyte-Derived Dendritic Cells (MDDCs)

10. **Day 0.** Thaw out cryovial(s) of frozen PBMCs by placing them in the 37°C water bath. Once partially thawed, add warmed media to the cell suspension and place into a 50 mL conical tube at a 1:9 ratio (1 mL cells:9 mL RPMI medium).
11. Centrifuge PBMCs at 500 × *g* at 20°C–22°C for 10 min. Decant supernatant and add 10 mL of fresh complete RPMI media. Repeat twice and count cells using a hemocytometer.
12. Resuspend PBMCs at 100 × 10^6^ cells/mL in EasySep Buffer and perform a positive selection for CD14+ monocytes using the EasySep human CD14 positive selection kit, following manufacturer’s protocol.
13. Resuspend monocytes at 1–2 × 10^6^ cells/mL in complete RPMI media supplemented with 50 ng/mL of rhGM-CSF and 50 ng/mL rhIL-4. Place cells in a T25 or T75 ultra-low attachment *NuncSphera* flasks and place in a 37°C/5% CO_2_ incubator.
14. **Day 2.** Collect and centrifuge cells at 400 × *g* for 10 min. Resuspend cells in fresh complete RPMI media + 50 ng/mL of rhGM-CSF and 50 ng/mL rhIL-4 and maintain cell concentrations at 1–2 × 10^6^ cells/mL. Return flasks to the incubator.
15. **Day 5.** Cells have differentiated into mostly immature DCs. Phenotypic analysis can be performed at this point, using anti-CD11c, CD80, CD86 and MHC-II antibodies where immature MDDCs are CD11c^+^CD80^low^CD86^low^MHC-II^low^. To obtain mature MDDCs, cells are resuspended at 0.8 × 10^6^ cells/mL in complete RPMI medium supplemented 25 ng/mL rhGM-CSF, 25 ng/mL rhIL-4 and 100 ng/mL of LPS (*S. Minnesota)* for 24 h. Phenotypic analysis by flow cytometry should be performed at this point to confirm their maturation, characterized by upregulation of CD80, CD86, and MHC-II expression.

***Note:*** The use of attachment free *Nunc Sphera* flasks is optional, but highly recommended to improve recovery of MDDCs by keeping cells in suspension. This removes the requirement for cell detachment solutions, such as using accutase, when harvesting cells for experiments and maintains high cell viability and yield.

***Note:*** Similar to naive T cell isolations, the viability and cell yield is high when using freshly isolated PBMCs compared to frozen cells. This is particularly the case for CD14+ monocytes, where their viability after freeze/thaw is lower compared to lymphocytes.

### Cell Labeling and Embedding into Collagen Matrix for Live-Cell Microscopy Studies

**Timing: 1–6 h for cell labeling and imaging in collagen gels**

Commercially available cell-labeling dyes are used to visualize cells in live-cell imaging studies within a fibrillar 3D collagen network that support robust immune cell migration.

#### Differential Labeling of T Cell and Dendritic Cells Using Celltracker Dyes

16. Count each cell type, centrifuge and resuspend in 1 mL of sterile cell staining buffer (1% FCS in PBS) at a maximum concentration of 10^7^/mL in a 15 mL conical falcon tube. Ideal amount of T cells and DCs to stain in a single tube is 4 and 8 million, respectively. If more cells need to be stained, use multiple falcon tubes.
17. The following cell tracker dye concentrations work well for T cells and dendritic cells, but should be titrated for other cell types to achieve the desired level of brightness.

**Table:** 

Reagent	Final staining concentration
Celltracker Blue (CMAC)	2 0μM
Celltracker Orange (CMTMR)	5 μM
Celltracker Green (CMFDA)	0.5 μM
Celltracker Deep Red (CMTPX)	0.25–0.5 μM

18. Make a 1 mL solution of Celltracker dye at 2× concentration with pre-warmed staining buffer and place in a 15 mL conical tube. Add 1 mL of cell suspension and mix well by pipetting up and down.
19. Place cells in a 37°C water bath for 15–20 min. To remove excess dye after incubation, underlay cell suspension with 2 mL of ice-cold 100% FCS, resulting in two distinct liquid phases (Figure 2).

**Figure 2:**
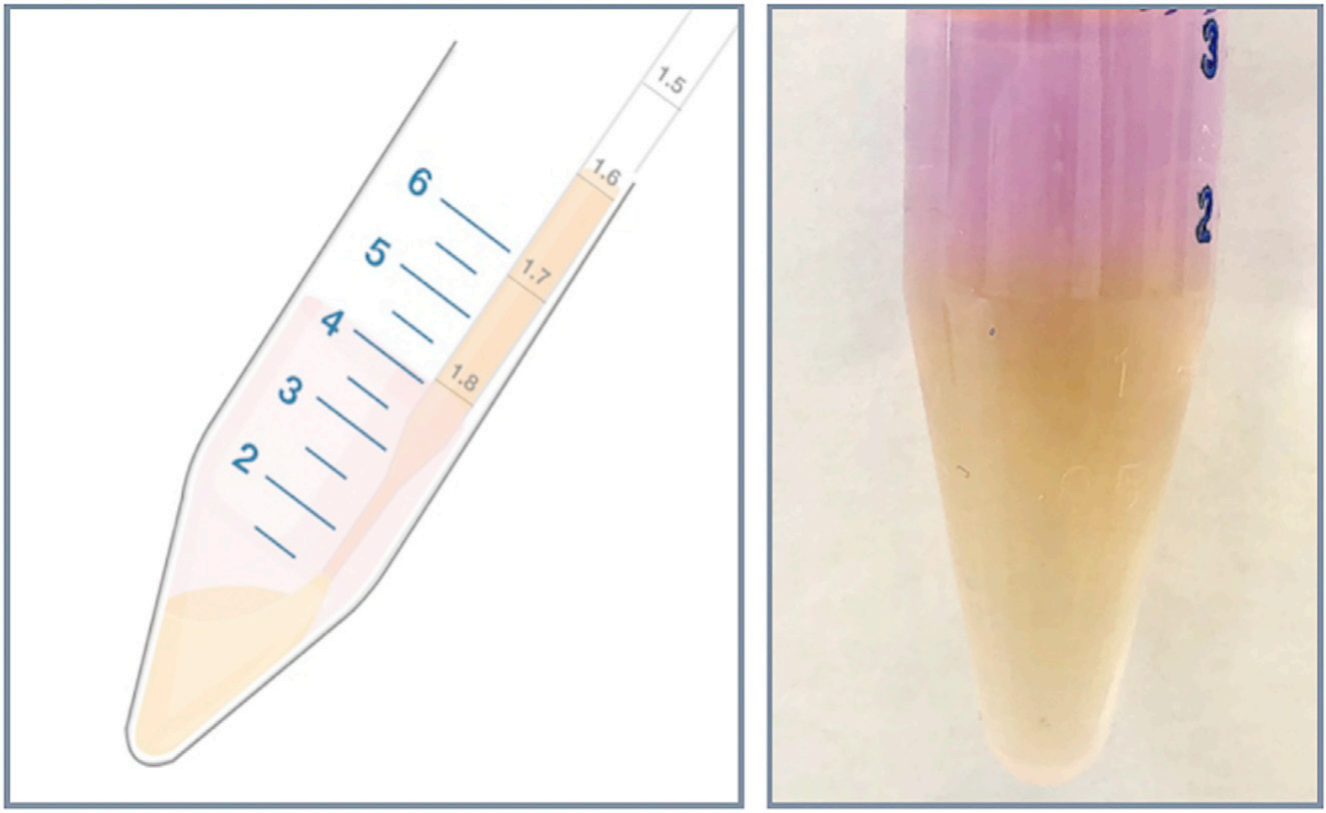
Removal of Excess Dye after Staining Left: underlaying cells with 100% FCS. Right: representative image of the two liquid phases (red labeled cells, top; and the FCS layer, bottom). Centrifuging cells through the FCS layer quenches excess dye and removes them from solution.
20. Centrifuge cells at 400 × *g* for 5 min. Aspirate out the culture supernatant without disturbing the cell pellet. Resuspend in 10 mL of complete RPMI media and centrifuge again at 400 × *g* for 5 min.
21. Prior embedding into collagen, these cells were kept in the complete RPMI supplemented with either GMCSF/IL-4 or IL-2 at 37°C for 30–60 min.

***Optional:*** cells can be incubated in complete RPMI for 20–60 min to remove any excess dyes instead of passing them through the FCS solution.

***Note:*** There are numerous commercially available cell dyes with a wide fluorescence spectrum that can be utilized for each microscopy setup. Some dyes will label specific cell structures, such as the cell membrane (CellLight Plasma-Membrane GFP [Thermo Fisher] or CellMask Deep Red [Themo Fisher]) or the nucleus (NucSpot [Biotium] or Biotracker Nuclear Dye [Sigma]), that can be helpful when identifying cell structures in real-time.

#### Construction of Gel Chambers and Embedding Cells into Collagen Matrices

22. Assemble the chamber slides by placing the adhesive silicone isolators (1 mm thickness) onto a microscope glass slide. Peel the adhesive from the top and adhere a 18 × 18 mm cover slip, leaving a 2–3 mm gap at one end. Ensure that there are no bubbles/holes for the collagen mixture to leak from (Figure 3).

**Figure 3:**
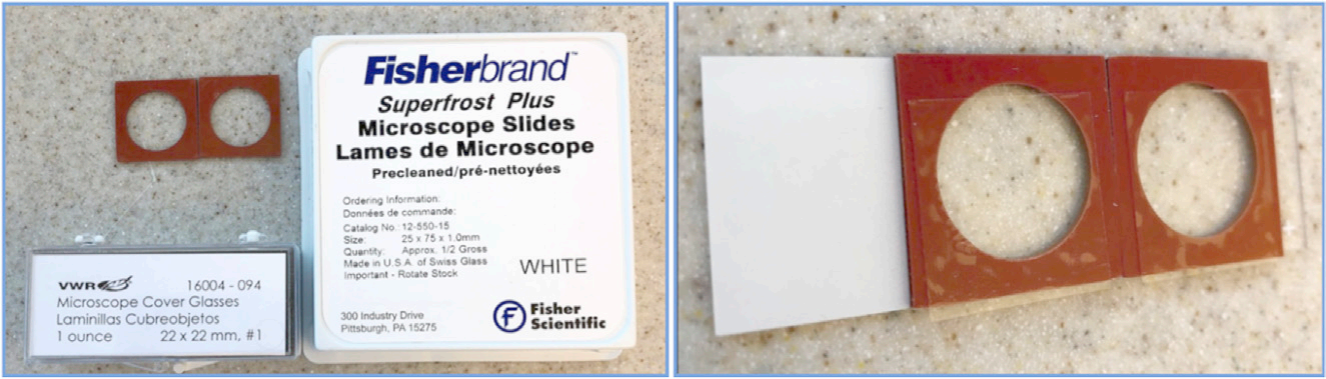
Construction of a Collagen Chamber Left: materials used. Right: up to two chambers can be placed on a single microscope slide.
23. Prepare collagen gel mixture in a 1.5 mL Eppendorf or 5 mL FACS tube.

**CRITICAL:** All reagents are stored in 4°C fridge and collagen-cell solution is quickly mixed at 20–22°C with cold reagents to prevent premature gel solidification.

**Table:** Bovine collagen gel (1.7 mg/mL)

Reagent	Final Concentration (mM or μM)	Amount
10× MEM	0.74×	20 μL
7.5% sodium bicarbonate	0.28%	10 μL
3.1 mg/mL Bovine PureColl	1.7 mg/mL	148 μL
Cells in complete RPMI		92 μL
**Total**	**n/a**	**270 μL**

24. Mix the collagen solution by pipetting up and down, careful not to introduce bubbles (Figure 4C). Add the cell suspension and mix without forming bubbles (Figure 4D). Carefully pipette into each slide chamber through the gap created with the cover slip.

**Figure 4:**
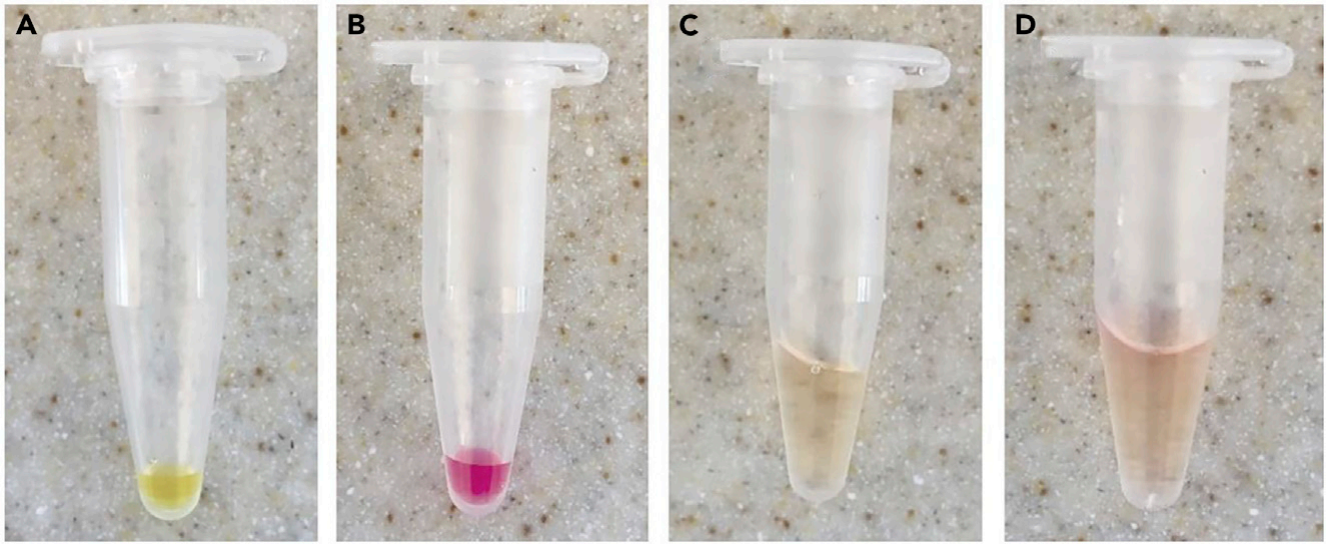
Representative Images of Collagen-Cell Mixture Preparation (A) 10× MEM solution. (B) MEM + sodium bicarbonate. (C) Addition of collagen solution. (D) Final collagen-cell solution.
25. Place the collagen chambers into the incubator (37°C, 5% CO_2_), standing the chamber up vertically and allowing gel to polymerize for 45 min – 1 h. Once solidified, collagen gels become opaque and has a pinkish orange color (Figure 5). Embedded cells are also visible under light microscope. Chambers are now ready for live-cell imaging studies.

**Figure 5:**
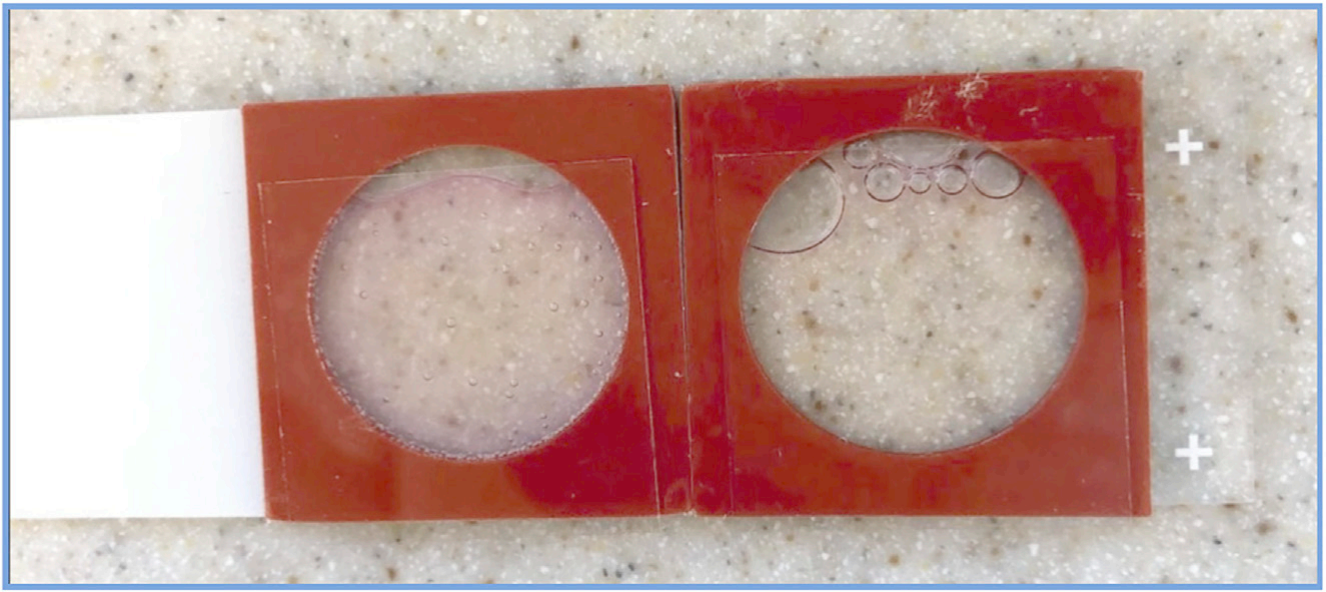
Image of a Properly Poured Collagen Gel Cells were embedded into collagen solution and poured into chambers. Left: properly solidified collagen matrix. Right: non-optimal collagen chamber that has not solidified and contains large bubbles.
26. Place collagen gel slides onto a heated platform and plug the heating resistors into the temperature controller. A thermocouple is placed into the gel to ensure temperature of the setup is maintained at 37°C (Figure 1).
27. Turn on two-photon femtosecond lasers and adjust to the optimal wavelength for maximal excitation of the Celltracker dye or fluorescent protein. We have found that Celltracker blue, green, orange, and red dyes are brightly excited at wavelengths between 780–830 nm. Images were acquired using a 20× water-immersion objective lens (Olympus, 1.0 N.A, 2.0 mm WD; XLUMPLFLN). Two-photon microscope allows for maximal imaging depth and less phototoxicity/photodamage to cells compared to conventional confocal microscopy.
28. Select image acquisition parameters and record time-lapse images. Typical parameters used for evaluating DC:T cell interactions are: 13 z-stacks at 4 μm spacing, 30 s per cycle, 121 total cycles. The length of movies depends the migratory behavior of T cells and DCs, as DCs migrate a much slower rate than T cells (Methods Video S1). Acquisition parameters should be optimized for each experiment to balance acquiring sufficient data and potential cytotoxic effects from repeated imaging.

***Note:*** Cells can be transduced with lentivirus-expressing fluorescent proteins as an alternative to fluorescent dyes. Advantage of this approach is that specific proteins and/or organelles can be visualized. Isolating cells from fluorescent reporter transgenic mice will eliminate the need for labeling cells prior to imaging studies.

***Note:*** Optimal voltage settings on the temperature controller should be determined for each heating platform prior to the experiment. A thermocouple should be used to ensure temperature of the collagen matrix gels are maintained at 37°C ± 0.5°C by inserting the probe into the gel. Humidity or CO_2_ control is not required for this collagen chamber setup.

Methods Video S1. Visualizing DC:T Cell Contact Dynamics in Collagen (Related to Step 28) Mature MDDCs (green) and activated CD4 T cells (blue) isolated from a healthy blood donor were embedded into collagen gels prepared for live-cell imaging. Time stamp are in minutes:seconds. Scale bar, 20 μm.

### Data Analysis for Cell Motility, Morphology, and Cell-Cell Interaction Dynamics

**Timing: 1–2 weeks**

**Figure 6:**
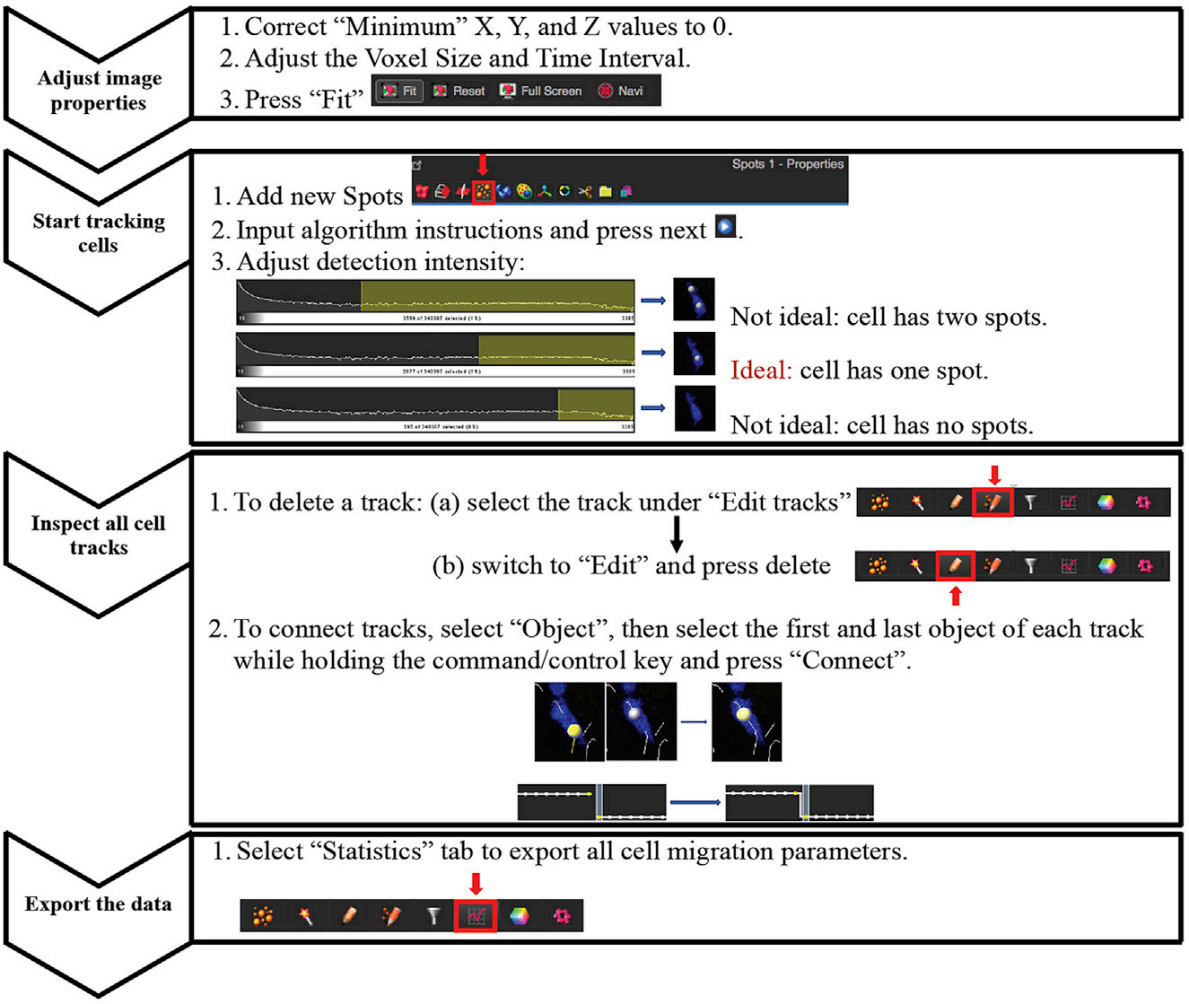
Imaris Analysis Flowchart Step-by-step flowchart describing how to analyze cell tracks with Imaris software.

Time-lapse microscopy images will be processed using available 4D image rendering software to quantitatively analyze cellular migration parameters. Comparison of cellular behaviors and cell-cell interaction dynamics can describe biological processes and responses. Here we used Imaris 8.2 software to track T cells and DC migration behaviors in 3D. The below protocol is meant to be a basic approach to track cells, and to highlight some issues with tracking (Figure 6). For a more complete set of instructions, please refer to the Imaris built-in program and operation manual.

#### Image Processing and Cell Migration Analysis Using Imaris 8 Software

29. Convert the original .xml file into .ims file using the Imaris FileConverter Software.
30. Open the .ims file in the Imaris 8.2 software.
31. Open **Image Properties** under the **Edit tab** and correct minimum X, Y, and Z values to 0. Adjust the **Voxel size** for the z value used to acquire the images (e.g., 4 μm). Under the **Time Point section**, select the **“All Equidistant” tab** and adjust the **Time Interval** to the experimental time period. If the image disappears, **press “Fit”** at the bottom left corner of the program (Figure 6).
32. To start tracking T cells over time, select **“Add new Spots”** (Orange circles).
33. Input algorithm instructions and press next. Select a small region of interest to implement the algorithm, which channel to analyze, and the approximate XY diameter.
34. Adjust the detection intensity by sliding the highlighted bar to the left or right to ensure that only one cell is spotted at any one time. It is also important to ensure each spot locates at the cell body. Click next.
35. Cell tracks where the entire cell is not in the field of view should be deleted from the analysis. Select the cell track in question by selecting the **Edit tracks** tab.
36. Inspect each cell tracks and ensure that they are accurately depicting the same cell over time. Separate tracks belonging to the same cell can be manually connected by selecting **Object** and selecting two objects (depicted as yellow balls) while holding the command/control key and pressing **“Connect.”** A single cell track after combining two separate tracks is shown below.

**CRITICAL:** it is important to visually inspect all cell tracks for accuracy. Migration analysis of DCs and T cells are done separately.

37. Once cell tracking is complete, select the **statistics tab** to export all cell migration parameters as an excel file. Further cell migration parameter analyses(Beltman et al., 2009) can be done using a custom script using MATLAB software, and will be made available upon request.

### 3D Immunohistochemistry in Collagen

**Timing: 7–8 days for embedding, staining, and imaging of 3D collagen blocks**

Cells of interest are embedded into 3D collagen gels, fixed *in situ* and prepared for immunohistochemistry (IHC). This approach preserves the 3D morphological features of migrating cells, allowing studies into protein expression/localization profiles in polarized cells.

#### Cell Embedding, Fixation, and Preparation for IHC

38. Embed cells of interest into collagen as detailed above. We recommend preparing 540 μL of cell:collagen mixture with no more than 3 million total cells (2 × 270 μL recipe above).
39. Dispense 540 μL of cell:collagen suspension into a 24 well plate, to create a cell bubble. Place the culture plate in the 37°C incubator to solidify.
40. After the desired incubation time, add 2 mL of pre-warmed 4% paraformaldehyde in 5% sucrose solution to each well and leave in the incubator for 24 h.

**CRITICAL:** Using fixation buffer warmed to 37°C for 30 min, and quickly adding it to collagen gels while in the incubator is important to maintain cell polarity. Any delay at this step will result in rounded cells.

41. Remove PFA solution and wash the gel gently with 2 mL of PBS containing 0.2% Tween-20, three times for 5 min per wash. Carefully aspirate the washing buffer.
42. Add 2 mL of 0.15 M glycine buffer (pH 7) to quench excess PFA by placing it in the 4°C fridge for 18–24 h. Make sure the gels are completely submerged in the glycine buffer.
43. Rinse gels with wash buffer (PBS containing 0.2% Tween-20) three times and carefully aspirate using a pipette.
44. To permeabilize the cells prior to staining, add 2 mL of 0.5% Triton X solution for 48 h in the fridge. Rinse gels three times with wash buffer and carefully aspirate using a pipette.
45. A blocking step is performed to reduce non-specific binding by antibodies. Add 2 mL of 1% bovine serum albumin (BSA) in PBS and leave for 18–24 h in the 4°C fridge. Rinse gels with wash solution three times and proceed to staining.

#### Staining for F-Actin Using Fluorescently Conjugated Phalloidin

**Figure 7:**
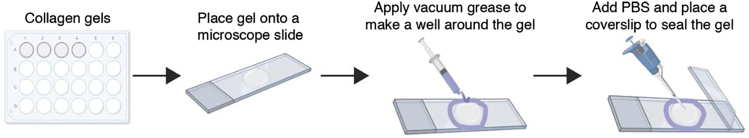
Workflow to Mount Collagen Gels for Microscopy Illustration was created with BioRender.com.
51. Place the mounted gels under the microscope and collect 3D stack images. We recommend taking z-stack spacing of 1–2 μm for 3D cell reconstruction. Images can be imported into Imaris software, as described above or other image analysis software (e.g., ImageJ) for expression/localization analysis.

***Note:*** This approach is amenable for staining using fluorescently conjugated antibodies or cell expressing fluorescent proteins such as GFP or RFP. If secondary antibodies are used to improve the signal-to-noise ratio, rinse gels five times, 5 min per wash between addition of primary and secondary antibody.

46. Transfer each collagen gel blocks into a new 24-well culture dish using a plastic spoon, and add 300–500 μL of 1.65 μM Texas red conjugated phalloidin solution (to cover entire gel), prepared according to manufacturer’s recommendation. Incubate the gels for 24 h in the 4°C fridge in the dark.
47. Rinse gels with wash buffer three times, 5 min per wash. Gently aspirate buffer with a pipette.
48. Using a plastic spoon, place the gels onto a glass microscope slide. Remove as much liquid as possible using a Kimwipe so that the gel remains in place.
49. Surround each gel with sufficient vacuum grease (dispensed using a 5 mL syringe) to create an airtight seal. Apply enough grease to match the height of the gel itself.
50. Place a small amount of PBS into the grease well and gently place a coverslip on top of the gel, slightly pushing down on the grease ring to form a seal (Figure 7). The preparation should be free of any large air bubbles: to remove these, inject additional PBS using an insulin syringe.

## Expected Outcomes

### Dynamic Visualization of DC:T Cell Interactions on Fibrillar Collagen Networks

**Figure 8:**
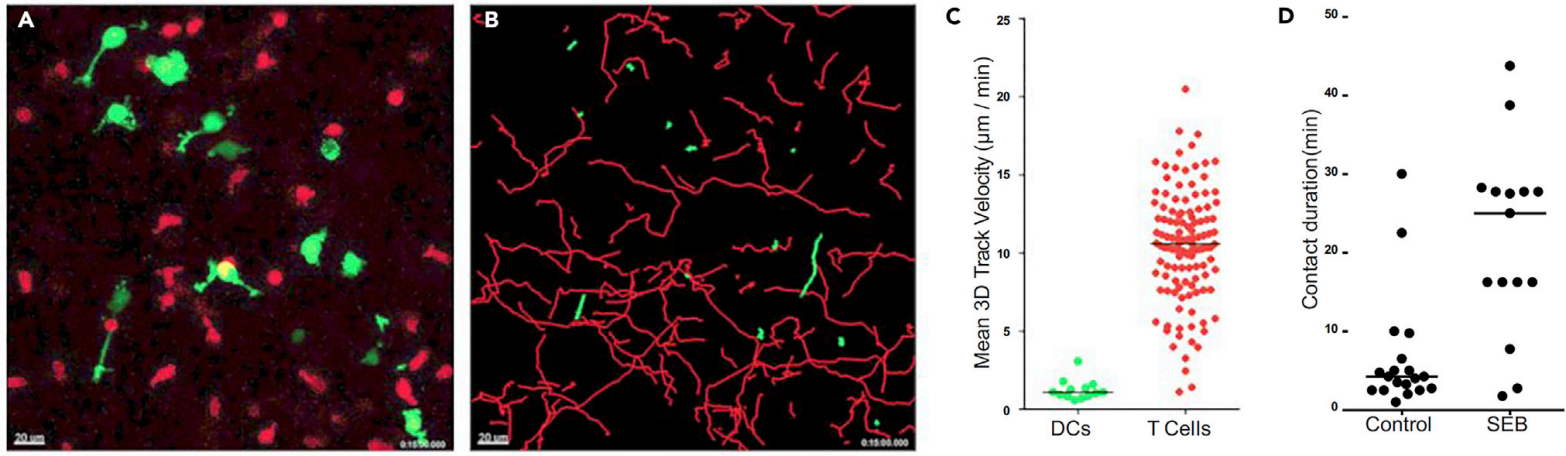
Representative Live-Cell Microscopy of DC:T Cell Interactions at Steady State in Collagen (A) Syngeneic DCs (green) and activated T cells (red) were co-embedded into collagen gels and visualized by live-cell microscopy. Scale bar, 20 μm. (B) DC and T cell tracks over the 30-min recording are shown. (C) Mean 3D track velocities of DCs (green) and T cells (red). Black lines depict median values. (D) DC:T cell contact duration of control or in the presence of *Staphylococcus* enterotoxin B (SEB). Black lines depict median values.

**Figure 9:**
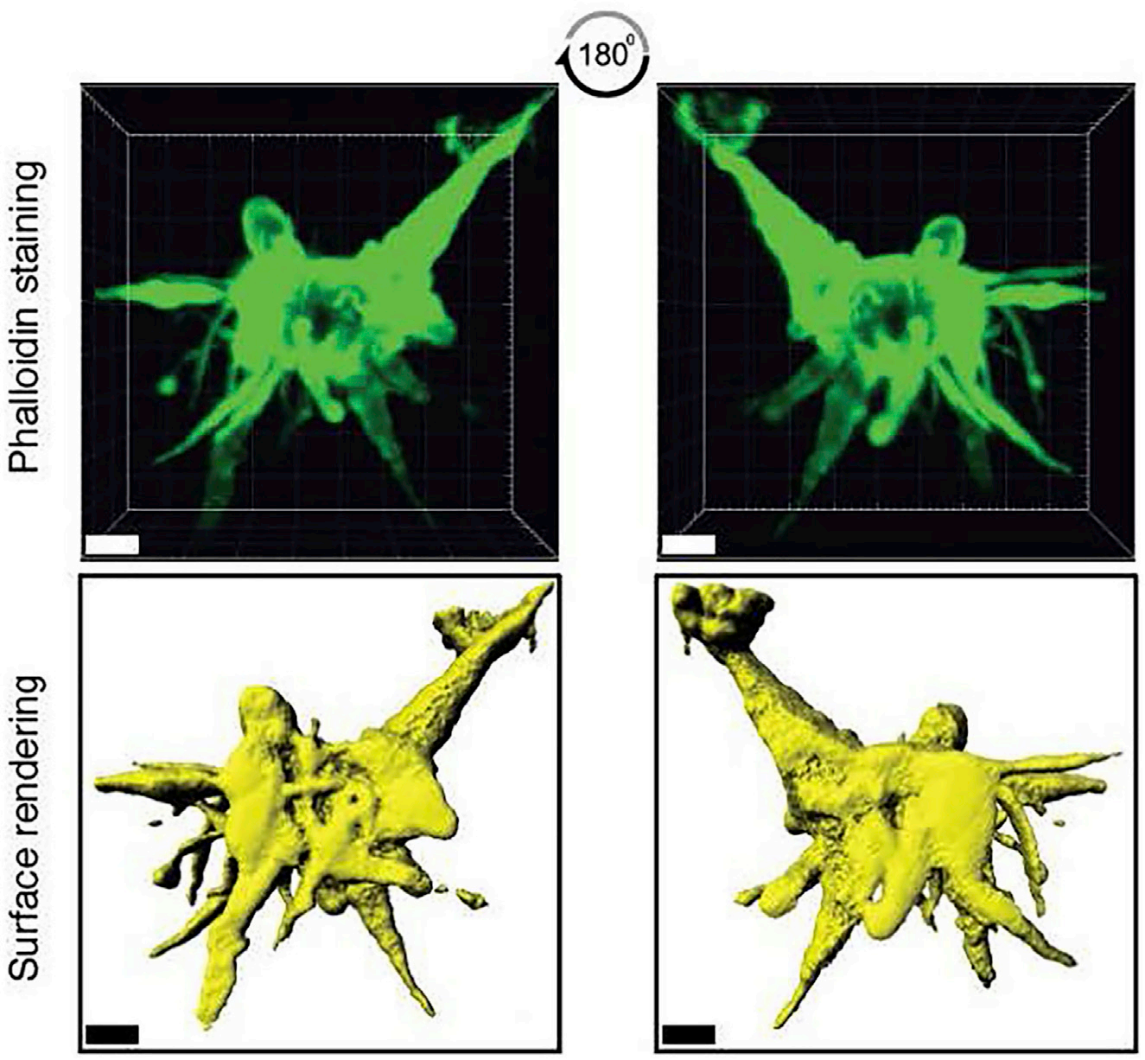
3D Reconstruction of a Mature DC Stained with Phalloidin in Collagen 180° rotation of the DC is shown to the right. F-actin staining helps visualize dendritic extensions. Bottom: 3D surface rendering using Imaris software. Scale bar, 5 μm.

Dendritic cells are important tissue sentinels that respond to microbial products and inflammatory stimuli and coordinate the development of adaptive T cell responses. Within secondary lymphoid organs, DC:T cell contacts occur at near-continuous frequencies along fibroblastic reticular cell (FRC)-ensheathed collagen bundles, and remain transient and non-productive in the absence of cognate antigen (Bousso and Robey, 2003, Miller et al., 2004). However, once antigen recognition occurs, prolonged DC:T cell interactions ensue and dictate the magnitude of T cell differentiation, effector function and memory responses (Henrickson et al., 2008, Mempel et al., 2004). Live-cell imaging studies in collagen gels provide researchers with a simple, reductionist *in vitro* model to study dynamic DC:T cell interactions in 3D. Collagen gels can be fixed immediately after imaging studies and evaluated for specific protein expression/localization *in situ* to complement findings from live-cell imaging studies. In Figure 8, physiological behaviors of human DCs and T cells in collagen are shown. Activated T cells migrate robustly at high migration speeds, whereas mature DCs are slowly motile (Figures 8A–8C; Methods Video S1). DC:T cell contact are transient at steady state, but become measurably prolonged in the presence of the superantigen SEB, as expected (Figure 8D). In Figure 9, mature DCs were stained with Texas red conjugated phalloidin and visualized *in situ*. F-actin drives the formation of dendritic extensions, which are visible and preserved in this preparation. Together, we present an updated approach to quantitatively assess physiological cell motility and cell-cell contact dynamics within a 3D setting, which can be used to describe how various stimuli or pathogens can impact such behaviors and effector responses.

## Limitations

Live-cell microscopy studies require healthy cells to acquire consistent and reliable cell migration measurements. Tissue culture conditions need to be optimized to ensure that only viable cells are used for imaging studies. Visualizing rare events, such as cell division, will require longer imaging durations which can impact cell viability. Careful monitoring of cell death or photobleaching of fluorophores is needed, and if observed, those frames should be removed from the final analysis. It is important to note that while the described reductionist model allows for migration studies in the absence of confounding factors, *in vivo* tissue environments not considered in these studies can impact cell functions, such as cytokine gradients and the presence of stromal or other immune cells.

## Troubleshooting

### Problem 1

Collagen gels do not solidify or melts during imaging during step 25.

### Potential Solution

The pH of the cell-collagen preparation may not be optimal. The PureColl solution has a pH of 2.0 and needs to be neutralized with sodium bicarbonate prior to adding cells. Use a litmus indicator strip to make sure the pH of the cell-collagen mixture is7.0 ± 0.2. If not, adjust the sodium bicarbonate volume to achieve optimal pH before embedding cells. We have also found that non-optimal pH leads to melting of the collagen gels during imaging.

### Problem 2

High background signals are observed during IHC analysis in collagen.

### Potential Solution

As is the case for standard immunohistochemistry analysis of tissue sections, poor signal-to-noise ratio makes it difficult to interpret results. Depending on the cell type being studied, background staining with either antibodies or dyes can vary. In this protocol, we block non-specific binding using a 1% BSA solution, but other blocking protocols can be used such as serum of the species in which the secondary antibody is derived from and Fc blocking antibodies/peptides. Prolonging the washing step may also lower background signals.

### Problem 3

Poor labeling of cells during the IHC preparation.

### Potential Solution

This may be caused by insufficient permeabilization of cells in collagen gels. In this protocol, gels are permeabilized with 0.5% Triton X solution for 48 h, but longer permeabilization times may be required for thicker gels (>1 mm thickness). Ensure that the solution containing primary/secondary/dyes completely covers the entire gel.

### Problem 4

Excessive cell death is observed after labeling with fluorescent dyes.

### Potential Solution

Although we do not observe significant cell death during our described staining protocols, some cell types are more sensitive to repeated manipulations, such as neutrophils and macrophages. The use of other commercially available cell-labeling dyes has the potential to have more toxicity. An alternative to use protein dyes is to perform lentiviral transduction with fluorescent reporters, or to isolate cells from reporter transgenic mice. Determining the right dye concentration and labeling times will be critical to achieve brightly stained cells with minimal death for live-cell imaging studies.

### Problem 5

Difficulty in tracking individual cells or due to excessive photobleaching.

### Potential Solution

Bias in the cell tracking analysis occurs when cells are very crowded in each field of view, causing migration tracks of two overlapping cells to converge. This can particularly become a problem with crowding of fast-moving cells, such as T cells, neutrophils and NK cells. If this is the case, lower the number of cells embedded into the gel, or increase gel volume to achieve a larger surface area. Thicker silicone isolators (e.g., 2 mm) are available from the manufacturer to accommodate more gel volume. We have found that photobleaching is an issue particularly with non-motile cells, because they are continuously exposed to exciting lasers. Labeling cells with higher concentration of dyes may help, but toxicity issues may arise. Increasing the laser scanning cycle period (e.g., taking an image every 45 or 60 s instead of 15 s) or reducing resolution may be strategies to mitigate excessive photobleaching issues.

## Resource Availability

### Lead Contact

Further information and requests for resources and reagents should be directed to and will be fulfilled by the Lead Contact, Thomas T. Murooka, thomas.murooka@umanitoba.ca

### Materials Availability

This study did not generate unique reagents.

### Data and Code Availability

Cell migration parameter analysis was performed using Imaris (Bitplane), ImageJ (NIH) and MATLAB (MathWorks). Custom MATLAB scripts will be made available upon request.
